# A portable system for rapid bacterial composition analysis using a nanopore-based sequencer and laptop computer

**DOI:** 10.1038/s41598-017-05772-5

**Published:** 2017-07-18

**Authors:** Satomi Mitsuhashi, Kirill Kryukov, So Nakagawa, Junko S. Takeuchi, Yoshiki Shiraishi, Koichiro Asano, Tadashi Imanishi

**Affiliations:** 1 Biomedical Informatics Laboratory, Department of Molecular Life Science, Tokai University School of Medicine, Isehara, Kanagawa 259-1193 Japan; 2 Division of Pulmonary Medicine, Department of Medicine, Tokai University School of Medicine, Isehara, Kanagawa 259-1193 Japan

## Abstract

We developed a portable system for 16S rDNA analyses consisting of a nanopore technology-based sequencer, the MinION, and laptop computers, and assessed its potential ability to determine bacterial compositions rapidly. We tested our protocols using a mock bacterial community that contained equimolar 16S rDNA and a pleural effusion from a patient with empyema, for time effectiveness and accuracy. MinION sequencing targeting 16S rDNA detected all 20 of the bacterial species present in the mock bacterial community. Time course analysis indicated that the sequence data obtained during the first 5 minutes of sequencing (1,379 bacterial reads) were enough to detect all 20 bacteria in the mock sample and to determine species composition, consistent with results of those obtained from 4 hours of sequencing (24,202 reads). Additionally, using a clinical sample extracted from the empyema patient’s pleural effusion, we could identify major bacterial pathogens in that effusion using our rapid sequencing and analysis protocol. All results are comparable to conventional 16S rDNA sequencing results using an IonPGM sequencer. Our results suggest that rapid sequencing and bacterial composition determination are possible within 2 hours after obtaining a DNA sample.

## Introduction

Time is of critical importance when identifying pathogens in acute infectious disease. In some critical conditions, such as bacteremia, starting antibiotic administration within 1 hour is highly recommended^1^. However, rapid pathogen identification is usually difficult, as conventional bacterial culture techniques require several days, yet alone most bacteria and fungi are difficult or impossible to grow in culture at all. Therefore, the initial choice of antibiotic is usually empirical. Establishing systems for rapid microorganism identification via metagenomic sequencing seems pertinent and practical, especially for clinical use, to facilitate appropriate initial antibiotic treatment.

Most currently available, next-generation sequencing (NGS) technologies are not designed for the rapid acquisition of sequence data^2^. NGS is usually based on several parallel fluorescence/proton scanning runs that obtain huge amounts of nucleotide sequence data, but require days to weeks to complete. However, a portable USB sequencer MinION (Oxford Nanopore Technologies, Oxford, UK) produces nucleotide sequence data sequentially, enabling real-time metagenomic analysis. Moreover, MinION has further advantages including simple sample preparation, portability, quick turnaround, maneuverability, and being relatively inexpensive. MinION has been in use for nearly 3 years since its release in 2014. A major update in its chemistry from R7 to R9, with a consequent dramatic improvement in sequencing accuracy, was accomplished in May 2016. MinION’s rapid library preparation protocol was also released in May 2016, which enables 10-min library preparation from DNA to bacterial identification. This protocol only reads the template strand of double-stranded DNA (1D sequencing), and was initially considered to be of relatively low quality. In contrast, the original library preparation protocol produced data from both strands (2D sequencing), but required 90 min for library preparation. The new R9 chemistry improves the usability of the data obtained from rapid 1D sequencing, generating high accuracy base calls. This novel NGS sequencing technology is increasingly being used for detecting pathogens in bacterial infections^3^.

Bacterial identification data analysis is also time-consuming and is currently often not feasible in many hospitals or diagnostic laboratories. *Centrifuge* is a novel microbial classification software that enables rapid and accurate identification of species, and can even be run on laptop computers^4^. However, it is not clear what combination of these novel technologies is necessary to provide a sufficiently quick and reliable bacterial detection system in a clinical setting.

Here, we show that the combination of rapid sequencing using transposase-mediated library preparation for nearly full-length 16S rDNA amplicons, rapid 1D sequencing using R9 chemistry, 5-min sequencing data acquisition, local base-calling, and rapid analysis using *Centrifuge* on a laptop computer enables us to determine major bacterial lineages within 2 hours, even in a small laboratory environment.

## Results

### Time-course analysis of MinION Rapid 1D sequence

**Figure 1:**
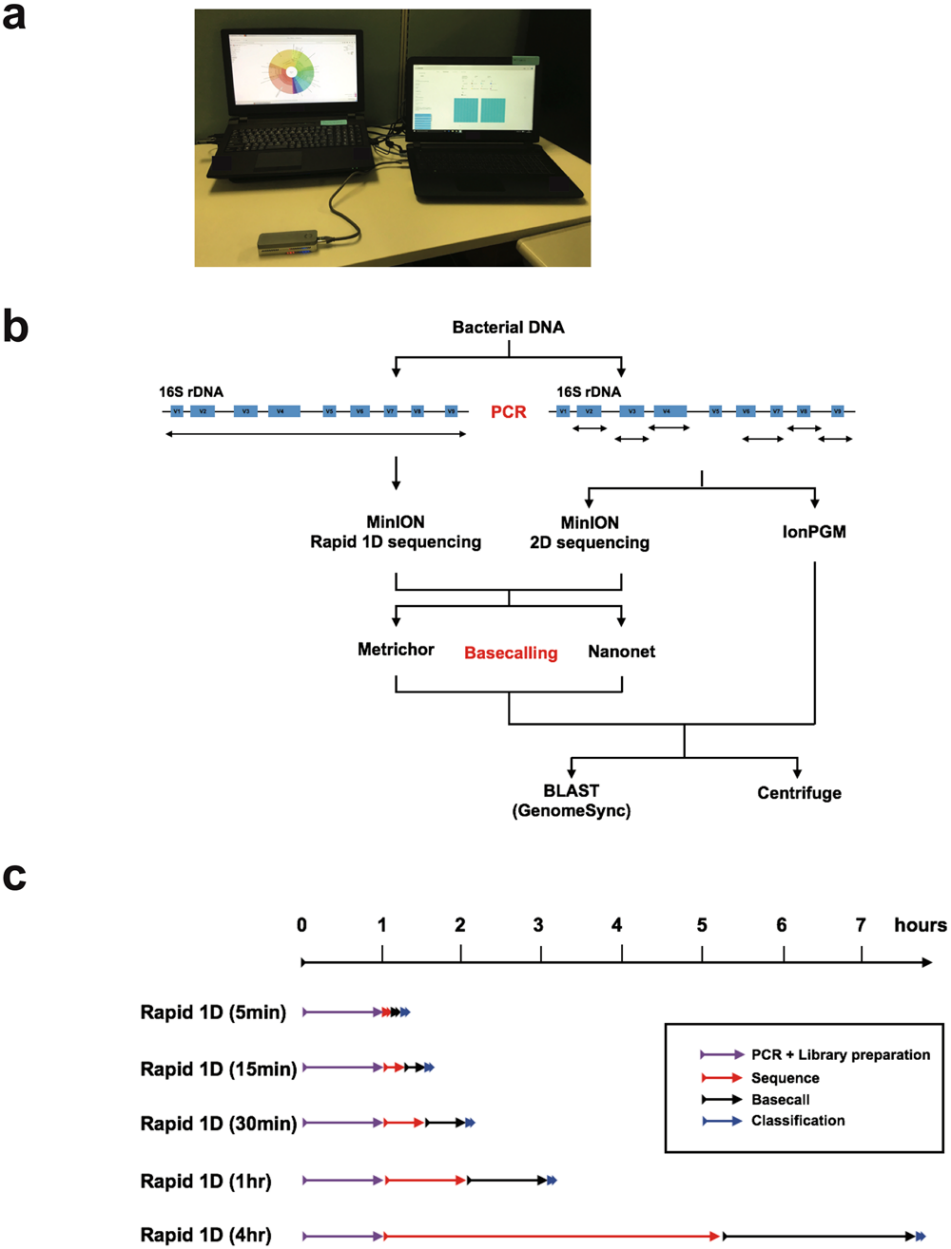
Portable system for bacterial determination and overview of our study. (**a**) Our system for bacterial composition determination. (**b**) Schematic outline of our experiment protocols. We used primer sets that amplified the same region of V2, V3, V4, V6–7, V8, and V9 for MinION 2D and IonPGM sequencing. For Rapid 1D sequencing, we amplified nearly the full-length of the16S rRNA genes. Metrichor and Nanonet software were used for base-calling. Results were analysed both with *Centrifuge* and BLAST-based searching methods. (**c**) Rapid 1D sequencing data at different time points, 5 min, 15 min, 30 min, 1 h, and 4 h, were collected and analysed to assess time-effectiveness. Figure shows the time course after sample DNA preparation. Detailed time count for “PCR + Library preparation” is as follows: PCR reaction, 41 minutes; DNA purification, 10 minutes; and library preparation using Rapid Sequencing Kit, 10 minutes.

We sequenced a mock bacterial community consisting of 20 species (Supplemental Table 1) using various protocols, to test species detection accuracy against a variety of bacteria. The mock bacterial community contained bacterial genomic DNA at a ratio of equal 16S rDNA copy numbers for each species that we could obtain from the Biodefense and Emerging Infectious Research (BEI) Resources as a mixture^5^. Therefore, we expected to obtain the same number of reads for each bacteria species present, if PCR efficiency is equal for all species. Rapid 1D sequencing using SQK-RAD001 (Oxford Nanopore Technologies, Oxford, UK) is designed for fast library preparation, and utilises transposase to fragment DNA, simultaneously attaching the necessary sequencing adapters to its free ends. This is unsuitable for short amplicons; therefore, we amplified nearly full-length 16S rDNA (1,399 bp) for MinION Rapid 1D sequencing (Fig. 1b, Supplemental Tables 2 and 3)^6^. We also tested our primer sets with 11 different bacterial species or strains that we obtained from BEI Resources (Supplemental Table 6). All 11 bacteria were also confirmed as amplified by our primer sets (Supplemental Figure 3A and B). Furthermore, MinION 2D sequencing and IonPGM (Thermo Fisher Scientific, MA, USA) sequencing were performed on the targeted 16S rDNA variable region sequences from these samples (Fig. 1b, Supplemental Table 2).

Sequencing data at five different time points (5 min, 15 min, 30 min, 1 h, and 4 h) from the beginning of MinION sequencing were used (Fig. 1c), to compare different sequencing methods, and assess time effectiveness (Fig. 1b). The differences observed between MinION 1D and 2D results may primarily be explained by the difference between the sequencing methods (1D sequencing reads only the template strand while 2D sequencing reads both strands) and/or by the difference in amplicon lengths (Fig. 1b, Supplemental Table 4).

**Figure 2:**
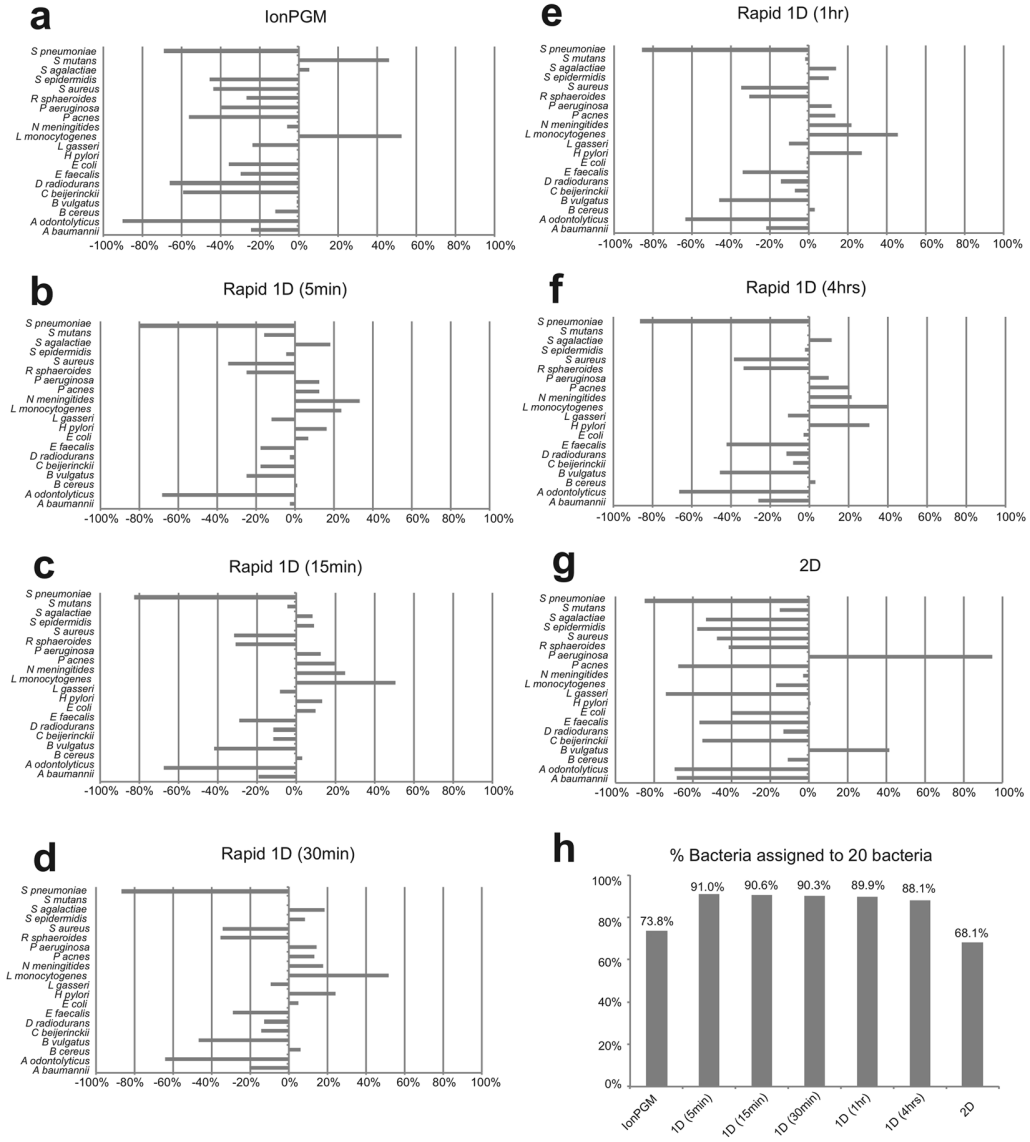
Species level bacterial classifications using the Nanonet base-caller and BLAST-based searches. Deviation from the percentage of expected reads to all reads assigned to bacteria are shown (**a**–**g**). IonPGM sequencing for 16S rDNA (**a**). MinION Rapid 1D Sequencing for near full-length 16S rDNA amplicons for 5 min (**b**), 15 min (**c**), 30 min (**d**), 1 h (**e**), and 4 h (**f**). MinION 2D sequencing for 16S rDNA (**g**). The percentage of reads assigned to any of the 20 bacteria among all reads classified as bacterial reads (**h**). The remaining percentage represents the taxa that were not assigned to the original mock community.

**Figure 3:**
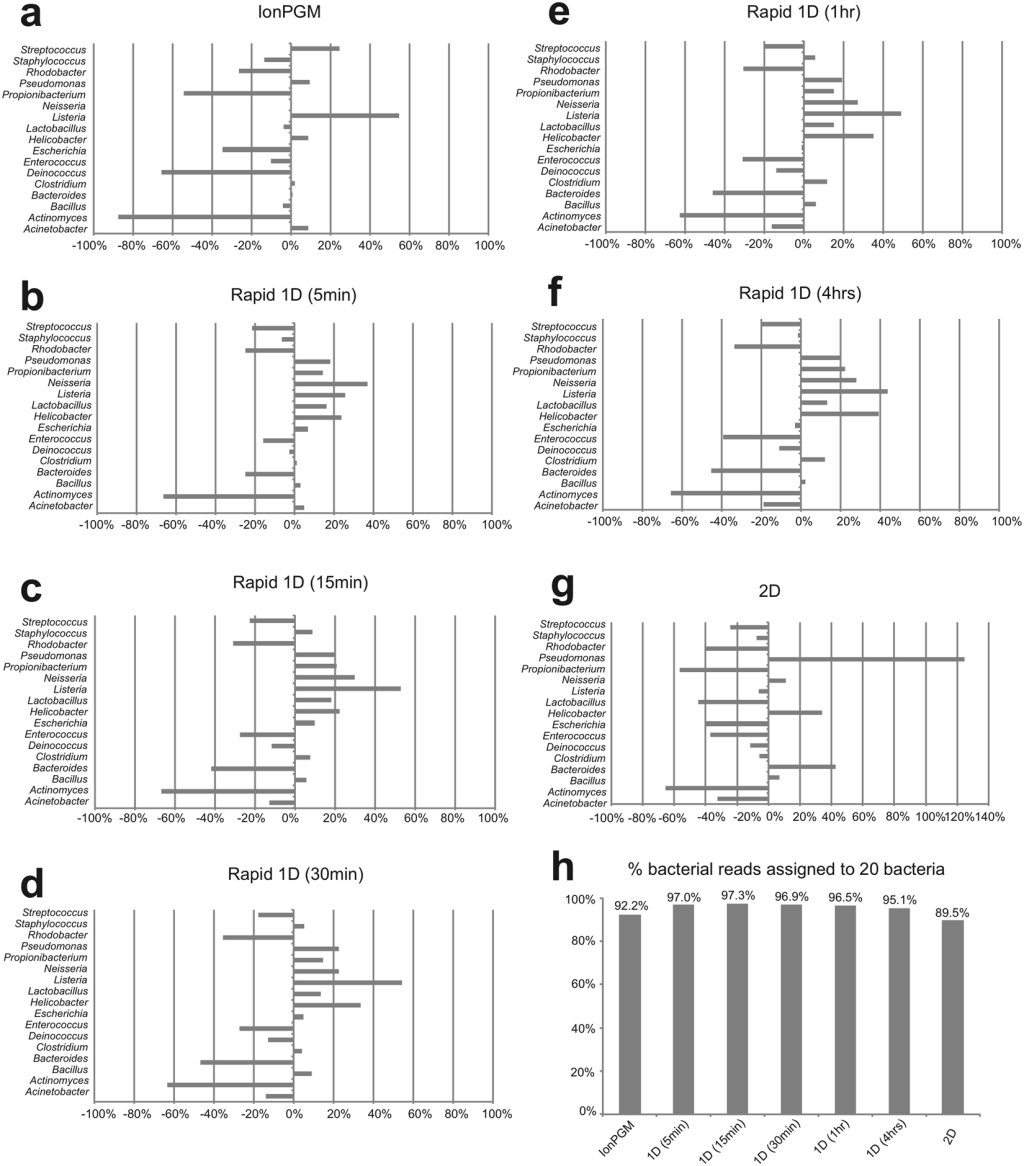
Genus level bacterial classifications using the Nanonet base-caller and BLAST-based searches. Deviation from the percentage of expected reads to all the reads assigned to bacteria are shown (**a–g**). IonPGM sequencing for 16S rDNA (**a**). MinION Rapid 1D Sequencing for near full-length 16S rDNA amplicons for 5 min (**b**), 15 min (**c**), 30 min (**d**), 1 h (**e**), and 4 h (**f**). MinION 2D sequencing for 16S rDNA (**g**). The percentage of reads assigned to any of the 20 bacteria among all reads classified as bacterial reads (**h**).

Initially we used the local base-calling software Nanonet for the MinION sequencing data. The resulting nucleotide sequences were searched using BLAST^7^ against our in-house genome database, GenomeSync (http://genomesync.org). Overall, we found that all sequencing protocols could identify all 20 bacterial species in the reference mock community at both the species and genus level (Figs 2a–f and 3a–f). We defined sensitivity in our study to correspond to that fraction of the number of reads mapped onto the 20 bacterial species or the corresponding 17 genera in the reference mock community. MinION 1D sequencing data showed the highest sensitivity and smallest deviation from the expected read number, versus the IonPGM or 2D sequencing data, at the species and genus levels (Figs 2a–f and 3a–f, Supplemental Table 5). Interestingly, 5 min of sequencing data consisting of 1,379 reads were comparable to those results from 4 h of sequencing generating 24,202 reads. (Figs 2h and 3h, Table 1). To examine this observation statistically, we compared the proportions of bacteria detected by MinION 1D 5-min sequencing data with those by 5-min–4-hour sequencing data, both at the genus and the species levels, using Spearman’s rank correlation. The resulting bacterial compositions of the 5-min and 5-min–4-hour sequencing runs were consistent with each other, at the genus and species levels, as analysed by BLAST/GenomeSync [R = 0.87 (p < 0.00001) and R = 0.83 (p < 0.00001), respectively]. The compositions were also comparable both at the genus and species levels when analysed by *Centrifuge* [R = 0.90 (p < 0.00001) and R = 0.88 (p < 0.000001), respectively. Rapid 1D sequencing for 5 min showed 91% and 97% sensitivity at the species and genus levels, respectively (Figs 2h and 3h, Supplemental Table 5), while 4 h of sequencing showed 88.1% and 95.1%, respectively. Overall, sensitivity is higher at the species level compared with the genus level. This suggests that a large proportion of misclassified reads are actually classified into closely-related taxa. These data collectively indicate that even 5 min of sequencing data can obtain bacterial compositions similar to those of longer sequencing runs.

**Table 1:** Time scale of each protocol.

Sequencer	Method	Reads*	Time							
			Amplicon preparation	Sequencer		Data analysis				
				Sequencer preparation	Sequencing	Ion Reporter	Base call by Metrichor and Poretools	Basecall by Nanonet	Centrifuge	BLAST
IonPGM	Ion Metagenomic kit	330,124	6 hr	~3 hr	3 hr	5–6hr	NA	NA	<1 min	NA
MinION	Rapid 1D (5 min)	1,379	10 min	20 min	5 min	NA	38 min	7 min	<20 sec	NA
MinION	Rapid 1D (15 min)	3,703	10 min	20 min	15 min	NA	101 min	19 min	<20 sec	NA
MinION	Rapid 1D (30 min)	6,906	10 min	20 min	30 min	NA	174 min	34 min	<20 sec	NA
MinION	Rapid 1D (1hr)	11,174	10 min	20 min	1 hr	NA	273 min	58 min	<20 sec	NA
MinION	Rapid 1D (4hr)	24,202	10 min	20 min	4 hr	NA	659 min	141 min	<20 sec	NA
MinION	2D (6 primer set)	348,973	90 min	20 min	48 hr	NA	4373 min	NA	<30 sec	NA

*Number of fast5 files for MinION. Note that this does not necessarily mean reads count.

We also tested nucleotide sequences base-called by Metrichor (https://metrichor.com), which uses cloud-computing services through the Internet (Fig. 1b). In the Metrichor base-called data, 5-min Rapid 1D sequencing results showed 86% and 94% sensitivity at the species and genus level, respectively (Supplemental Figures 2H and 3H, Supplemental Table 5). Overall, Nanonet base-calling showed better sensitivity at the genus level for detecting the 20 bacteria with less deviation, compared with Metrichor (Supplemental Table 5). Furthermore, Nanonet is nearly five times faster than Metrichor (Table 1), although it requires relatively high CPU power.

### Centrifuge analysis

The BLAST-based search portion of classification analysis is time-consuming, even when using computer clusters. To reduce the computational time required for species detection, we tested a newly developed species classification suite, *Centrifuge* ^4^. At the species level, *Centrifuge* could identify all bacteria included in the mock community, except *Actinomyces odontolyticus*. On the other hand, at the genus level, all 20 bacteria (i.e. the corresponding 17 genera) were detected with a sensitivity of 63%, 68%, and 86% in the sequencing results of MinION Rapid 1D (5 min) and 2D, and IonPGM, respectively. We do not know why *Centrifuge* analysis could not detect *Actinomyces odontolyticus* at the species level; however, at the genus level, *Actinomyces* was detected in 23%, 29%, and 15% of the expected *Actinomyces* reads in Rapid 1D sequencing (5 min), 2D sequencing, and IonPGM, respectively. Therefore, the possibility exists that the actual composition of *Actinomyces* in our sample is lower than projected, despite the difference in methods of bacterial identification, primers used, and sequencers. Indeed, BLAST analysis also detected *Actinomyces* in 34%, 35%, and 12% of the expected reads in Rapid 1D sequencing (5 min), 2D sequencing, and IonPGM, respectively. Overall, *Centrifuge* exhibited less sensitivity and larger deviations, compared with BLAST-based analyses, but *Centrifuge*’s classification time was under 1 minute per run.

### Rapid 1D sequencing and analysis for pleural effusion derived DNA

**Figure 4:**
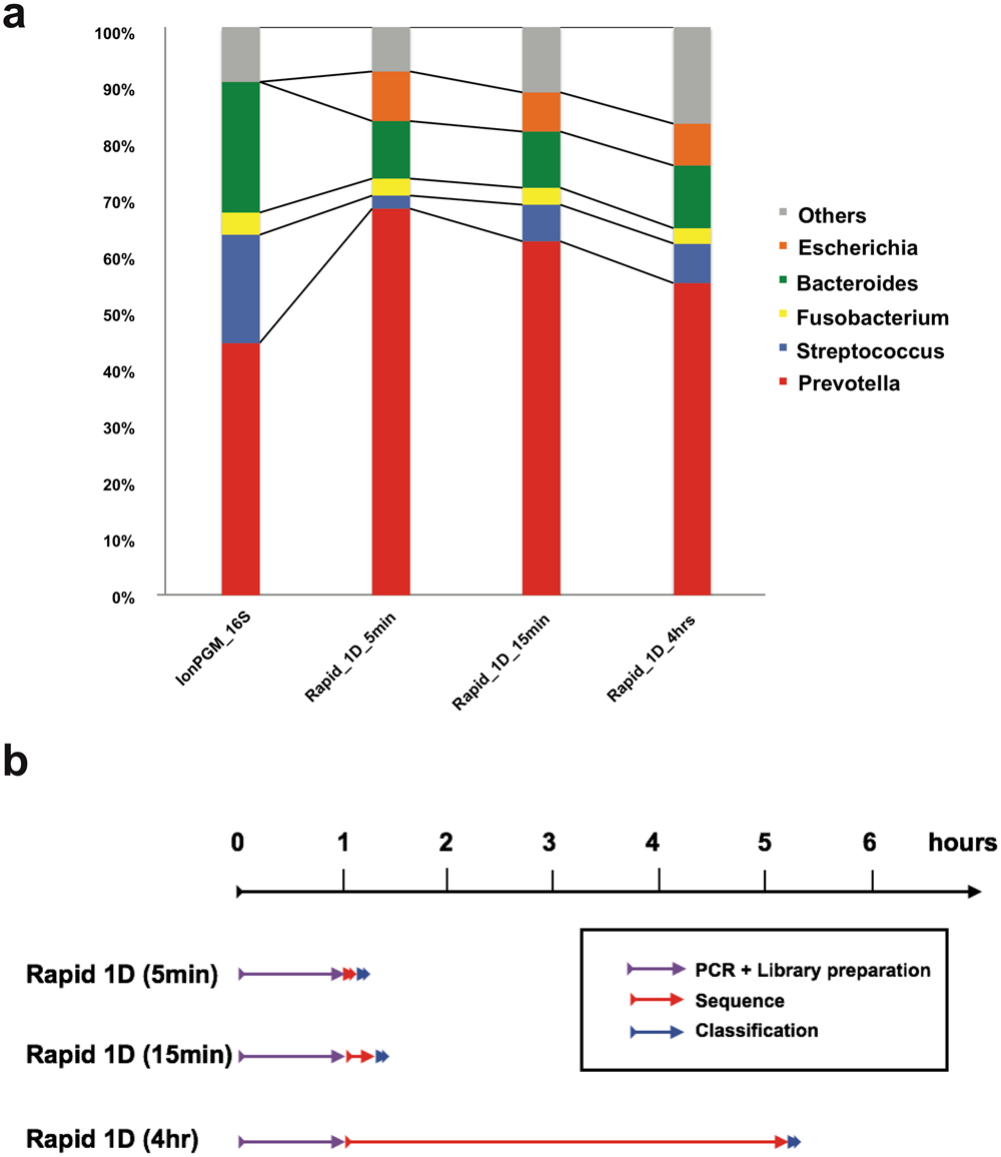
Clinical application of our system for determining bacterial composition in a pleural effusion from a patient with empyema. (**a**) Pleural effusion sample sequenced using three different methods indicating that *Prevotella* was the predominant bacteria. (**b**) Timescale for the experiment. Flow cell preparations took ~20 min for quality check and priming, which can be done during the PCR.

Finally, to examine whether this rapid sequencing protocol is applicable to clinical samples, we sequenced the total DNA samples extracted from the pleural effusion of a patient with empyema, in which microbiological examination identified the *Streptococcus anginosus* group and unculturable Gram-negative rods. We compared sequencing results between IonPGM sequencing and MinION rapid 1D sequencing. We also sequenced the same sample using IonPGM with 16S rDNA PCR amplification. All sequencing methods showed *Prevotella* as the major taxon in the pleural effusion from this patient (Fig. 4a). *Prevotella* was detected in 44%, and 68% of all the bacterial reads in IonPGM 16S rDNA amplicon sequencing, and MinION rapid 1D sequencing (5 min), respectively, suggesting that *Prevotella* was the major taxon in the sample. Supporting this conclusion, shotgun sequencing using an IonPGM sequencer also showed *Prevotella* as the major taxon (Supplemental Figure 8).

Prevotella are gram-negative bacteria in oral flora and are known to cause anaerobic infections of the respiratory tract, including empyema^8, 9^. Thus, we suspect *Prevotella* is the dominant bacteria that caused disease in this patient.

Bacterial culture suggests this clinical sample also contains *Streptococcus anginosus*. In concurrence, *Streptococcus* was also detected by all the sequencing methods we used. *Centrifuge* classifies 6.3% of total bacterial reads into the *S*. *anginosus* group, while 0.5% were classified as *S*. *anginosus* species in 4 hours. In addition, the fraction of *Streptococcus* increased to 2%, 6%, and 7% in 5 minutes, 15 minutes, and 4 hours, possibly suggesting that bacteria comprising a small fraction of a community may require longer sequencing times than 5 min to detect. *Centrifuge* provided the fastest classification with <20 sec for MinION data and <1 min for IonPGM data (Table 2). The total time from sample preparation to bacterial identification was within 2 hours (Fig. 2b).

**Table 2:** Time scale for the pleural effusion sample sequencing.

Sequencer	Method	Reads	Time						
			Amplicon preparation	Sequencer		Data analysis			
				Sequencer preparation	Sequencing	Ion Reporter	MinKNOW Base call and poretools*	Centrifuge	BLAST
IonPGM	Ion Metagenomic kit	429,974	~6 hr	~6 hr	3 hr	5–6 hr	NA	<1 min	NA
MinION	Rapid 1D (5 min)	1,012	10 min	20 min	5 min	NA	<20 sec	<20 sec	NA
MinION	Rapid 1D (15 min)	3,523	10 min	20 min	15 min	NA	<20 sec	<20 sec	NA
MinION	Rapid 1D (4hr)	54,544	10 min	20 min	4 hr	NA	<1 min	<20 sec	NA

*Base-calling was performed simultaneously while sequencing using MinKNOW software.

Further, detailed analyses using BLAST^7^ against our GenomeSync database indicate that *Prevotella oris* was the major species in the sample. Our BLAST searches show the composition of *P*. *oris* to total bacterial reads to be 70% and 85%, in IonPGM 16S rDNA amplicon sequencing, and MinION rapid 1D sequencing (5 min), respectively. However, *Centrifuge* could not detect *P*. *oris*, but instead classified the reads as *Prevotella* genus only, because the genome from this species is not included in the *Centrifuge* genome index.

## Discussion

Performing rapid, on-site sequencing of clinical samples containing pathogenic bacteria for determining a first choice antibiotic regime would prove incredibly informative. Although our study is still in trial stages, it provides preliminary evidence that the species composition of a mock bacterial community can be successfully detected within 2 hours using MinION sequencing and data analysis on laptop computers. We performed data analysis with two different methods of bacterial classification: BLAST-based searching and *Centrifuge*. *Centrifuge* is very rapid, and could detect all the bacteria in our samples at the genus level; however, as our study revealed, it may misclassify some bacteria at the species level. Several factors contribute to this limitation, including the quality of MinION reads^10^, the size of the *Centrifuge* database, and *Centrifuge*’s limited ability to detect distant homology.

Although the time calculated here for computational analysis may differ from laboratory to laboratory because of computer performance, these data provide valuable insight for constructing a bacterial identification system for researchers. Our sequencing data from the reference mock bacterial community are available from the DDBJ DRA database (DRA005399) and may be useful for examining bacterial classification in varied environments.

Our results show that rapid 1D sequencing using amplicons that cover nearly full-length 16S rDNA has better sensitivity (91.0%) compared with sequencing protocols using short and multiple amplicons (68.1%) in BLAST-based analyses (Fig. 2h). This suggests that long read sequences from 1D sequencing are suitable for the identification of species. Supporting this, the percentage of our reads mapping to the 20 bacteria 16S rDNA sequence mock community reference library using LAST^11^ from 1D long amplicon sequencing (55.8–61.0%, Supplemental Table 8) is greater than that from 2D short and multiple sequencing (14.7%, Supplemental Table 8), while the percentage of matched bases is comparable (85.3–85.4% in 1D and 86.5% in 2D sequencing, Supplemental Table 8). As the MinION sequencer is capable of reading more than several thousand bases, it may be intriguing to target regions such as 23S rDNA, which is longer than 16S rDNA, or the entire rDNA operon, which may show even better resolution for bacterial identification.

We have shown a rapid and accurate method for bacterial identification based on 16S rDNA PCR amplification; however, 16S rDNA sequencing has limitations. For example, drug resistant bacterial strains are largely indistinguishable from related bacteria based on 16S rDNA sequences alone. Schmidt *et al*. reported the detection of bacteria drug-resistant genes by directly sequencing DNA from heavily infected urine samples using MinION^3^. They used bacteria-rich urine samples, and therefore, human DNA contamination was minimised. This method may be difficult to apply for bacteria-scarce samples, such as blood specimens from infected patients, as some reports have shown that this may require >30 million reads^12^. However, new sequencing technologies are continuously being developed with improved data size capabilities and accuracies. Sequencing bacteria-scarce clinical samples without PCR amplification may even be possible as MinION matures and its capacity improves in the near future.

## Conclusions

Our results suggest that the 2-hour rapid determination of bacterial composition using a MinION sequencer and laptop computer is feasible, and that the system and the protocol presented in this study may be applicable to clinical use as a diagnostic support tool in hospitals or small laboratories in the near future. Further improvements regarding computational bacterial identification methods may provide enhanced resolution at the species level.

## Materials and Methods

### Bacterial DNA

Genomic DNA from 20 different bacteria was obtained from the Biodefense and Emerging Infectious Research (BEI) Resources (http://www.beiresources.org) of the American Type Culture Collection (ATCC) (Manassas, VA, USA). Mock Microbial Community B (BEI catalogue number HM-782D) contains genomic DNA from 20 different bacterial strains with an equal molar quantity of 16S rDNA for each organism (equimolar ribosomal RNA operon counts; 100,000 copies per organism per µL)^5^. Double-strand DNA concentration was measured with a Qubit Fluorimeter using a dsDNA HS Assay kit (Thermo Fisher Scientific, MA, USA).

### 16S rDNA amplicons and PCR

The 16S primers used in this study are shown in Supplemental Table 2. Six sets of primers for short amplicon sequencing were designed for 16S rDNA variable regions (V2, V3, V4, V6–7, V8, and V9), for 2D sequencing. The pooled PCR product was subjected to 2D DNA library preparation. Primers S-D-Bact-0008-c-S-20 and S-D-Bact-1391-a-A-17, which cover nearly the full length of 16S rDNA, were used for 1D sequencing^13–15^. One nanogram of DNA was used for PCR. KAPA HiFi HotStart ReadyMix (KAPA Biosystems, MA, USA) and Agencourt AMPure XP system (Beckman Coulter Genomics, CA, USA) were used for PCR and PCR product purification, respectively. The PCR conditions are shown in Supplemental Table 3.

### IonPGM sequencing for 16S rDNA and data analysis

Bacterial 16S rDNA regions were amplified by PCR using two primer pools covering variable regions (V2, V3, V4, V6–7, V8, and V9) from the Ion 16S™ Metagenomics Kit (Thermo Fisher Scientific, MA, USA). Thereafter, emulsion PCR was performed using the Ion OneTouch™ System that was loaded onto an Ion 316™ Chip. It was sequenced using an Ion PGM™ with HiQ chemistry according to the manufacturer’s protocol (Thermo Fisher Scientific, MA, USA). Fastq files were generated by Ion Reporter System (Thermo Fisher Scientific, MA, USA), which were subjected to data analysis.

### Amplicon DNA library preparation and DNA sequencing using MinION

Library preparation was performed using a SQK-NSK007 Nano Sequencing Kit R9 version (Oxford Nanopore Technologies, Oxford, UK) and Rapid Sequencing Kit SQK-RAD001 (Oxford Nanopore Technologies, Oxford, UK) using 1 µg and 200 ng amplicon DNA, for 2D amplicon sequencing and rapid 1D sequencing, respectively, according to the manufacturer’s protocols. MinION sequencing was performed using the MinION Mk1 b sequencer and FLO-MIN104 flow cells. Raw data (fast5 files) were obtained using MinKNOW software ver. 1.0.2 (Oxford Nanopore Technologies, Oxford, UK).

### Data Analysis

Base-calling was performed using Nanonet and Metrichor software, both developed by Oxford Nanopore Technologies. Nanonet is a local (i.e., no Internet-connection required) base-caller based on recurrent neural network (RNN) algorithms, while Metrichor is a cloud-based software that uploads row output files produced by MinION (fast5 files) to its server and downloads base-called fast5 files. We used Nanonet for the 1D sequencing base-calls, with default parameters. We also used Metrichor’s 1D base-calling (FLO_MIN105 250bms) for 1D sequencing runs, and 2D Base-calling RNN (SQK-MAP007) for 2D sequencing runs. The quality scores, and conversion to FASTA format for the base-called Metrichor fast5 files, were generated using Poretools ver 0.6.0^16^.

### Time-course dependent bacterial identification using BLAST and *Centrifuge*

Rapid bacterial identification was performed with *Centrifuge* software using bacterial, viral, and human genome datasets^4^. The identification of bacteria was also accomplished using BLASTN^7^ search against the GenomeSync database (http://genomesync.org). Representative bacterial and fungal genomes, as well as the human genome were used. Taxa were determined using an in-house script, then visualised using Krona Chart^17^. We performed bacterial composition analysis on the MinION 1D sequence reads dataset with Nanonet and Metrichor during the first 5 min, 15 min, 30 min, 1 h, and 4 h from the time sequencing was started. We also compared the 1D sequencing results with those from MinION 2D sequencing and IonPGM 16S metagenomics sequencing.

### Estimation of MinION sequencing accuracy from LAST alignment

To estimate accuracy we counted the percentage of mismatches in gapped sequence alignments of MinION generated sequences. Alignment was done using LAST software^11^ to the 20 bacterial 16S rDNA reference sequences.

### Laptop Computers

Two laptop computers were used for the analyses. MinION sequencing and Metrichor base-calling were performed on one (OS, Windows 10; CPU, Intel Core i7 6700HQ; memory, 8 GB; storage, 960 GB SSD); and other was used for Nanonet base-calling, running Poretools, and bacterial identification by Centrifuge (OS, CentOS 7; CPU, Intel Core i7 6700 K; memory, 32 GB; storage, 1 TB SSD) (Fig. 1a).

### DNA preparation from the pleural effusion in a patient with empyema

A pleural effusion sample was collected from a 77-year-old, male patient with empyema for diagnostic purposes with written informed consent. This patient had a history of diabetes, cerebral infarction, and gingivitis. The collected sample was centrifuged at 50 × *G* for 10 min to remove human white blood cells, and was then re-centrifuged at 15,500 × *G* for 10 min to pellet the bacteria. After removing the supernatant, the cells were re-suspended with lysis buffer (20 mM TrisHCl, pH 8.0, 2 mM EDTA, 20 µg/µl lysozyme, 1.2% Triton X) at 37 °C for 30 min, and then subjected to DNA extraction using a DNeasy mini kit (Qiagen, Hilden, Germany) according to manufacturer’s protocol. Extracted DNA was used for IonPGM sequencing (Thermo Fisher Scientific, MA, USA), and MinION 1D sequencing using the Rapid 1D sequencing kit with S-D-Bact-0008-c-S-20 and S-D-Bact-1391-a-A-17 primers.

### Pleural effusion sample sequencing and analysis

MinION 1D sequencing was performed using the Rapid Sequencing Kit SQK-RAD001 (Oxford Nanopore Technologies, Oxford, UK) with 200 ng of amplicon DNA. The MinION Mk1 b sequencer and FLO-MIN105 flow cell were used for sequencing. We used MinKNOW software ver. 1.1.17 for this sequencing run. This version allows local base-calling for 1D sequencing data using the protocol script NC_48Hr_Sequencing_Run_FLO_MIN104_plus_1D_Basecalling, to minimise analysis time. Bacterial identification was performed using BLAST and *Centrifuge*, as described above, and results were compared between the two sequencers. IonPGM sequencing using the same DNA was performed using the above-mentioned 16S Metagenomics Kit (Thermo Fisher Scientific, MA, USA).

### Statistical analysis

Spearman’s Rank Correlation efficient test was performed using R software.

### Ethics

This study was carried out with the approval of the Institutional Review Board for Clinical Research, Tokai University School of Medicine (14R220). All the experiments were performed in accordance with the Laboratory Biosafety Manual published by the World Health Organization (3^rd^ Edition, 2004).

### Web Resources

The URLs for data presented are as follows: GenomeSync http://genomesync.org

### Data access

The 20 bacterial mock community data were submitted to the DDBJ DRA database under accession number DRA005399.

## Electronic supplementary material

Supplemental data
