# Supplementary material for: A portable system for rapid bacterial composition analysis using a nanopore-based sequencer and laptop computer
Source: Sci Rep. 2017 Jul 18;7:5657. doi: 10.1038/s41598-017-05772-5 (PMC5516037; doi:10.1038/s41598-017-05772-5)

A portable system for rapid bacterial composition analysis using a nanopore-based sequencer and laptop computer

Satomi Mitsuhashi[1, a], Kirill Kryukov[1, a], So Nakagawa[1, a], Junko S Takeuchi[1], Yoshiki Shiraishi[2], Koichiro Asano[2], Tadashi Imanishi*[1]

1. Biomedical Informatics Laboratory, Department of Molecular Life Science, Tokai University School of Medicine, Isehara, Kanagawa 259-1193, Japan
2. Division of Pulmonary Medicine, Department of Medicine, Tokai University School of Medicine, Isehara, Kanagawa 259-1193, Japan

a. Authors contributed equally

***Corresponding author**

Tadashi Imanishi

Biomedical Informatics Laboratory

Department of Molecular Life Science

Tokai University School of Medicine

143 Shimokasuya, Isehara, Kanagawa 259-1193, Japan

Tel. +81-463-93-1121

Fax. +81-463-93-5418

Email: imanishi@tokai.ac.jp

Supporting materials

Supplemental Table 1

Bacterial composition for the Mock Community

Supplemental Table 2

Primer sequence used for this study.

Supplemental Table 3

PCR conditions used for this study.

Supplemental Table 4

Sequence statistics for the 20 bacteria mock community data. Metrichor classified the reads into fail and pass. We used both fail and pass reads for the analysis.

Supplemental Table 5

Sensitivity was calculated as % bacteria assigned to 20 reference bacteria to total bacteria reads. Percent deviation was calculated as percent expected reads for certain bacteria to all the reads assigned to the bacteria. Average of deviation was calculated to compare the extent of deviation from expectation in each data. Sensitivity and deviation at the genus level are shown.

Supplemental Table 6

Eleven different bacteria species (strains) obtained from BEI resource used for determining PCR condition.

Supplemental Table 7

Sequence statistics for the pleural effusion data.

Supplemental Table 8

Difference of MinION sequencing data to the published reference 20 bacteria mock community 16S rDNA sequences.

Supplementary Figure 1

The bacterial classification at the species level using the Metrichor base-caller and BLAST-based searches. Deviation from % expected reads for certain bacteria to all the reads assigned to bacteria are shown (a-g). IonPGM sequencing for 16S rDNA (a). MinION Rapid 1D Sequencing of the almost full-length 16S rDNA amplicon for 5 min (b), 15 min (c), 30 min (d), 1 h (e), 4 h (f). MinION 2D sequencing for 16S rDNA (g). The percentage of reads assigned to any of the 20 bacteria among all reads classified as bacterial reads (h).

Supplementary Figure 2

The bacterial classification at the genus level using Metrichor basecaller and BLAST-based searches. Deviation from % expected reads for certain bacteria to all the reads assigned to bacteria are shown (a-g). IonPGM sequencing for 16S rDNA (a). MinION Rapid 1D Sequencing of the almost full-length 16S rDNA amplicon for 5 min (b), 15 min (c), 30 min (d), 1 h (e), 4 h (f). MinION 2D

sequencing for 16S rDNA (g). The percentage of reads assigned to any of the 20 bacteria among all reads classified as bacterial reads (h).

Supplementary Figure 3

Eleven different bacteria species (strains) obtained from BEI resource were amplified either using S-D-Bact-0008-c-S-20 and S-D-Bact-1391-a-A-17 primer set (a) or six primers covering V2, V3, V4, V6-7, V8 and V9 (b), using KAPA HiFi HotStart ReadyMix (KAPA Biosystems, MA, USA). Amplified 11 bacteria species (strains) are shown in Supplemental Table 6.

Supplementary Figure 4

The bacterial classification at the species level using Nanonet base-caller and Centrifuge. Deviation from % expected reads for certain bacteria to all the reads assigned to bacteria are shown (a-g). IonPGM sequencing for 16S rDNA (a). MinION Rapid 1D Sequencing of the almost full-length of the 16S rDNA amplicon for 5 min (b), 15 min (c), 30 min (d), 1 h (e), 4 h (f). MinION 2D

sequencing for 16S rDNA (g). The percentage of reads assigned to any of the 20 bacteria among all reads classified as bacterial reads (h).

Supplementary Figure 5

The bacterial classification at the genus level using Nanonet base-caller and *Centrifuge*. Deviation from % expected reads for certain bacteria to all the reads assigned to bacteria are shown (a-g). IonPGM sequencing for 16S rDNA (a). MinION Rapid 1D Sequencing of the almost full-length 16S rDNA amplicon for 5 min (b), 15 min (c), 30 min (d), 1 h (e), 4 h (f). MinION 2D sequencing for 16S rDNA (g). The percentage of reads assigned to any of the 20 bacteria among all reads classified as bacterial reads (h).

Supplementary Figure 6

The bacterial classification at the species level using Metrichor base-caller and *Centrifuge*. Deviation from % expected reads for certain bacteria to all the reads assigned to bacteria are shown (a-g). IonPGM sequencing for 16S rDNA (a).

MinION Rapid 1D Sequencing of the almost full-length 16S rDNA amplicon for 5 min (b), 15 min (c), 30 min (d), 1 h (e), 4 h (f). MinION 2D sequencing for 16S rDNA (g). The percentage of reads assigned to any of the 20 bacteria among all reads classified as bacterial reads (h).

Supplementary Figure 7

The bacterial classification at the genus level using Metrichor base-caller and *Centrifuge*. Deviation from % expected reads for certain bacteria to all the reads assigned to bacteria are shown (a-g). IonPGM sequencing for 16S rDNA (a). MinION Rapid 1D Sequencing of the almost full-length 16S rDNA amplicon for 5 min (b), 15 min (c), 30 min (d), 1 h (e), 4 h (f). MinION 2D sequencing for 16S rDNA (g). The percentage of reads assigned to any of the 20 bacteria among all reads classified as bacterial reads (h).

Supplementary Figure 8

Shotgun sequencing for pleural effusion sample DNA was performed according to the manufacture's protocol (Thermo Fisher Scientific, MA, USA). Briefly, 100 ng of DNA was fragmented using Covaris sonicator (Covaris, MA, USA) and was then subjected to library preparation using a fragmentation kit (Thermo Fisher Scientific, MA, USA). Adapter-ligated DNA fragment was size-selected using eGel (Thermo Fisher Scientific, MA, USA). We obtained total read number of 80115. Among then, 300 bacterial read was included as most of the reads are from human DNA.

	Species	Strain	Genome size	16S copy number	Taxonomy ID
1	*Acinetobacter baumannii*	5377	3,976,747	6	470
2	*Actinomyces odontolyticus*	1A.21	2,391,230	3	1660
3	*Bacillus cereus*	NRS 248	5,224,283	13	1396
4	*Bacteroides vulgatus*	ATCC® 8482ä	5,163,189	7	821
5	*Clostridium beijerinckii*	NCIMB 8052	6,000,632	14	1520
6	*Deinococcus radiodurans*	R1 (smooth)	2,648,638	2	1299
7	*Enterococcus faecalis*	OG1RF	2,739,625	4	1351
8	*Escherichia coli*	MG1655	4,641,652	7	562
9	*Helicobacter pylori*	26695	1,667,867	2	210
10	*Lactobacillus gasseri*	63 AM	1,894,360	6	1596
11	*Listeria monocytogenes*	EGDe	2,944,528	6	1639
12	*Neisseria meningitidis*	MC58	2,272,360	4	487
13	*Propionibacterium acnes*	KPA171202	2,560,265	3	1747
14	*Pseudomonas aeruginosa*	PAO1-LAC	6,264,404	4	287
15	*Rhodobacter sphaeroides*	ATH 2.4.1	4,131,542	4	1063
16	*Staphylococcus aureus*	TCH959	2,830,017	5	1280
17	*Staphylococcus epidermidis*	PCI 1200	2,499,279	6	1282
18	*Streptococcus agalactiae*	2603 V/R	2,160,267	7	1311
19	*Streptococcus mutans*	UA159	2,032,925	5	1309
20	*Streptococcus pneumoniae*	TIGR4	2,160,842	4	1313

Supplemental Table 1. BEI Resourse bacterial mock community

Primers	Primer sequence	Product size
V2_Foward	AGNGGCGNACGGGTGAGTAAC	
V3_Foward	ACTGAGACACGGTCCANACTCCTACGGG	230
V4_Foward	CCAGAGCCGCGGTAATACGNAG	
V6-7_Foward	ACAAGCGGNGGANCATGTGGTTTAATTCGA	280
V8_Foward	TGTCGTCAGCTCGTGTCG	
V9_Foward	GAGGAAGGTGGGGATGACGTC	270
V2_Reverse	CTGCCTCCCGTAGGAGTCTG	
V3_Reverse	GTATTACCGCGGCTGCTGGCAC	270
V4_Reverse	GGACTACCAGGGTATCTAATCCTGTT	
V6-7_Reverse	TTGACGTCATCCCCACCTTCCTCC	290
V8_Reverse	CGATTACTAGCGANTCCNNCTTC	
V9_Reverse	GTTACGACTTCACCCCANTC	330
S-D-bact-0008-c-S20	AGRGTTYGATYMTGGCTCAG	
S-D-bact-1391-a-A-17	GACGGGCGGTGWGTRCA	1384

Supplemental Table 2. Primer sequences used for this study

PCR condition for V2, V4, V8, V9

95℃	5min	
98℃	20sec	
59.5℃	15sec	25 cycle
72℃	5sec	
72℃	5min	

PCR condition for V3, V6-7

95℃	5min	
98℃	20sec	
62℃	15sec	25 cycle
72℃	5sec	
72℃	5min	

PCR condition for full length 16S

95℃	5min	
98℃	20sec	
60℃	15sec	25 cycle
72℃	15sec	
72℃	5min	

Supplemental Table 3. PCR conditions used in this study

basecaller	method	fail/pass	total reads	total base pairs	read length mean	read length median	read length min	read length max	N25	N50	N75
Nanonet	Rapid 1D (5min)	NA	1167	975842	836.20	804	9	6489			
Nanonet	Rapid 1D (15min)	NA	3170	2652458	836.74	801	5	6489			
Nanonet	Rapid 1D (30min)	NA	5733	4904350	855.46	810	5	9473			
Nanonet	Rapid 1D (1hr)	NA	9510	8144141	856.38	812.5	5	9473			
Nanonet	Rapid 1D (4hr)	NA	20541	17965589	874.62	813	5	9473			
Nanonet	2D	NA	125382	64189793	511.95	446	7	9455			
Metrichor	Rapid 1D (5min)	fail	263	208046	791.05	751	111	1507	1085	884	662
		pass	923	798052	864.63	830	133	2160	1186	959	734
Metrichor	Rapid 1D (15min)	fail	668	532385	796.98	747	111	8196	1099	894	668
		pass	2478	2157453	870.64	834	133	2241	1183	984	728
Metrichor	Rapid 1D (30min)	fail	1174	954386	812.94	767	111	8196	1108	904	689
		pass	4456	3930651	882.1	847	133	8089	1189	995	738
Metrichor	Rapid 1D (1hr)	fail	2003	1621719	809.65	782	111	8196	1104	904	693
		pass	7189	6355257	884.03	850	133	8767	1187	993	740
Metrichor	Rapid 1D (4hr)	fail	4873	4017117	824.36	787	111	26840	1121	917	695
		pass	14502	12802088	882.78	840	105	64458	1188	988	739
Metrichor	2D	fail	520645	228194084	438.29	297	5	382528	1134	572	325
		pass	205725	86904188	422.43	329	103	5827	649	487	308

Supplemental Table 4. Sequencing statistics

Sequcencer	Basecaller	Method	*Centrifuge*					BLAST/GenomeSync				
			Total reads	Total Bacterial Reads	Sensitivity (%)	Average % deviation from expectation for each genus	Absolute average deviation from expectation for each genus (%)	Total reads	Total Bacterial Reads	Sensitivity (%)	Average % deviation from expectation for each genus	Absolute average deviation from expectation for each genus (%)
IonPGM	NA	Ion 16S™ Metagenomics Kit	378324	377582	86.2	-18.0	39.8	330123	322213	92.2	-11.2	24.1
MinION	Nanonet	Rapid 1D (5min)	1257	1108	62.6	-34.9	37.2	1067	1167	97.0	-0.7	18.4
MinION	Nanonet	Rapid 1D (15min)	3247	2885	60.9	-36.1	39.0	2895	3170	97.3	-1.1	24.1
MinION	Nanonet	Rapid 1D (30min)	5751	5132	60.2	-36.9	40.3	5259	5733	96.9	-1.8	23.7
MinION	Nanonet	Rapid 1D (1hr)	9445	8469	58.9	-38.3	41.8	8642	9510	96.5	-2.1	23.9
MinION	Nanonet	Rapid 1D (4hr)	17268	19430	51.8	-38.7	42.4	18665	20541	95.1	-3.3	24.6
MinION	Nanonet	2D (6 primer set)	85779	84432	68.0	-29.5	45.2	125382	91475	89.5	-9.0	34.8
MinION	Metrichor	Rapid 1D (5min)	1236	1185	63.2	-34.5	40.0	1149	1186	94.0	-3.7	17.6
MinION	Metrichor	Rapid 1D (15min)	3333	3190	65.0	-33.2	36.8	3066	3146	94.0	-4.1	22.6
MinION	Metrichor	Rapid 1D (30min)	5731	6014	61.2	-34.1	36.4	5500	5630	93.9	-4.8	22.6
MinION	Metrichor	Rapid 1D (1hr)	9237	9694	60.8	-34.1	37.2	8969	9192	93.6	-4.7	23.9
MinION	Metrichor	Rapid 1D (4hr)	19031	19903	59.3	-35.6	38.5	18908	19376	93.1	-5.2	24.7
MinION	Metrichor	2D (6 primer set)	93417	92289	68.0	-30.1	42.9	139784	121282	89.4	-10.8	31.0

Supplemental Table 5. Sensitivty to detect 20 mock bacteria and deviation from expected read number at genus level.

	Species	strain
1	*Staphylococcus aureus*	MRSA131
2	*Staphylococcus aureus*	HFH-29568
3	*Staphylococcus aureus*	HFH-30106
4	*Escherichia coli*	BAA-1161
5	*Streptococcus pneumoniae*	TCH8431
6	*Campylobacter jejuni*	UA466
7	*Bacillus cereus*	G9241
8	*Bacillus samanii*	C1
9	*Enterococcus faecalis*	TX0104
10	*Enterococcus faecalis*	TUSoD11
11	*Listeria monocytogenes*	10403S

Supplemental Table 6. Eleven bacterial species used for PCR condition determination.

					read length						
sequencer	method	basecaller	total reads	total base pairs	mean	median	min	max	N25	N50	N75
MinION	Rapid 1D (5min)	MinKNOW	670	421945	629.77	545	127	9002	1032	718	507
MinION	Rapid 1D (15min)	MinKNOW	2179	1447755	664.41	562	39	15219	1128	770	538
MinION	Rapid 1D (4h)	MinKNOW	38976	31298407	803.02	614	39	170904	1291	861	603

Supplemental Table 7. Sequence statistics for Pleural effusion sample.

Sequence Method	total reads	mapped reads	% mapped read	mismatches	gap opens	query bases	matched bases	% matched to query	% gap appears
Rapid 1D (5min)	1,186	723	61.0	10,499	3,005	71,800	61,301	85.4	4.2
Rapid 1D (15min)	3,146	1,905	60.6	27,677	7,964	189,004	161,327	85.4	4.2
Rapid 1D (30min)	5,630	3,366	59.8	48,015	13,696	326,975	278,960	85.3	4.2
Rapid 1D (1hr)	9,192	5,385	58.6	76,649	21,931	522,039	445,390	85.3	4.2
Rapid 1D (4hr)	19,376	10,813	55.8	150,275	43,765	1,028,280	878,005	85.4	4.3
2D (6 primer set)	726,370	107,021	14.7	992,704	225,930	7,347,232	6,354,528	86.5	3.1

Supplemental Table 8. Difference to published reference 20 bacteria genomes

Supplemental Figure 1

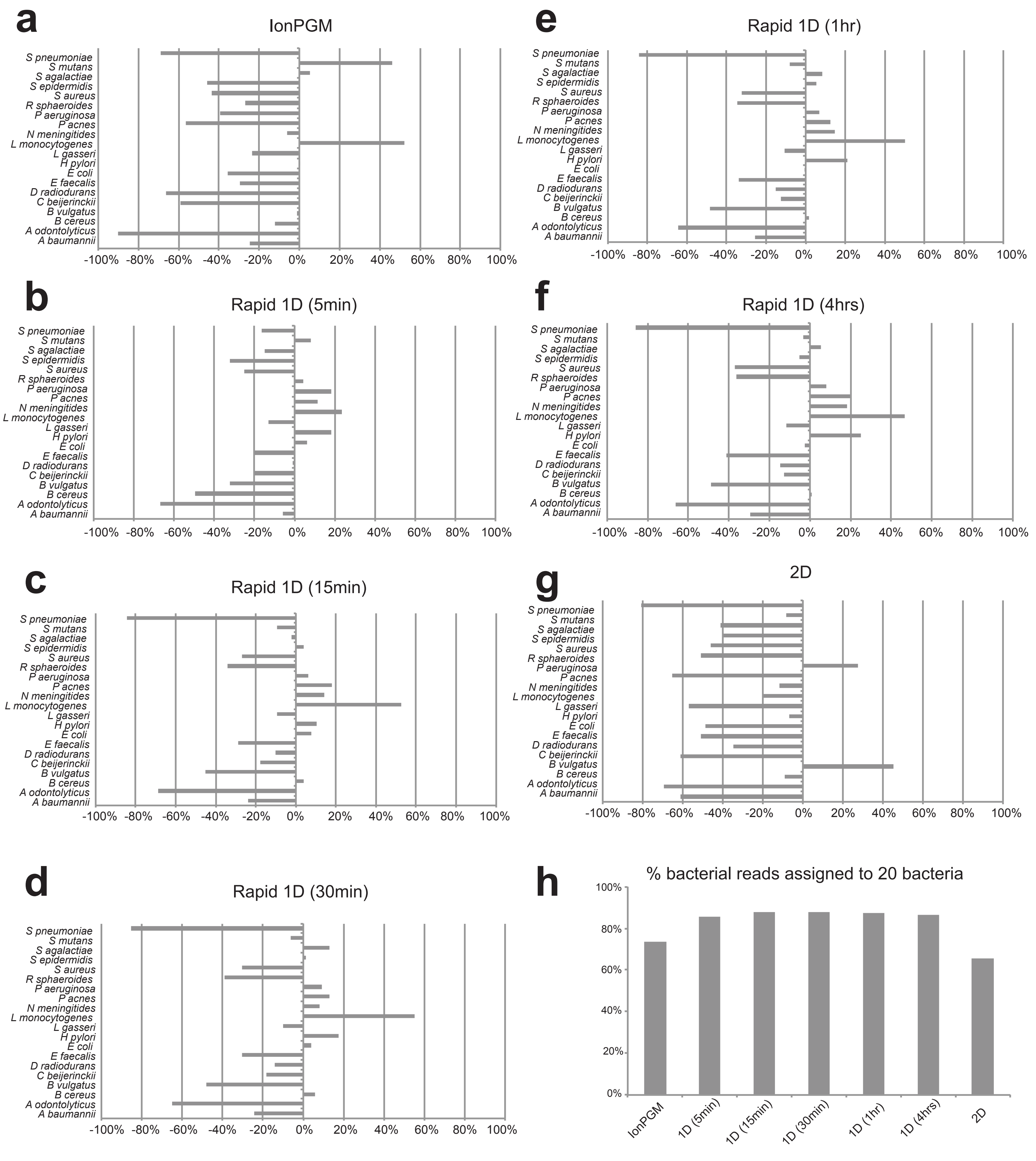

Supplemental Figure 2

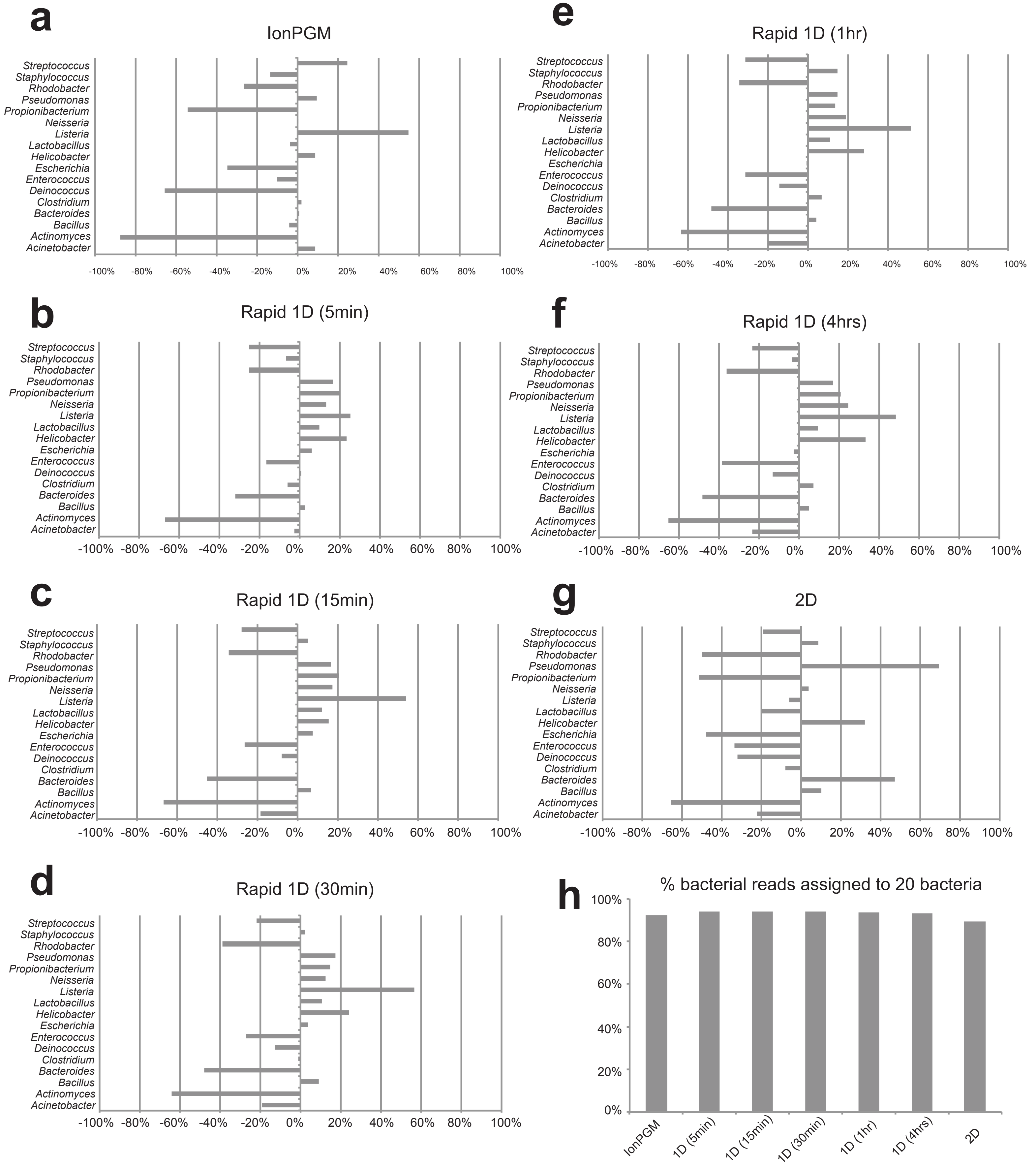

Supplemental Figure 3

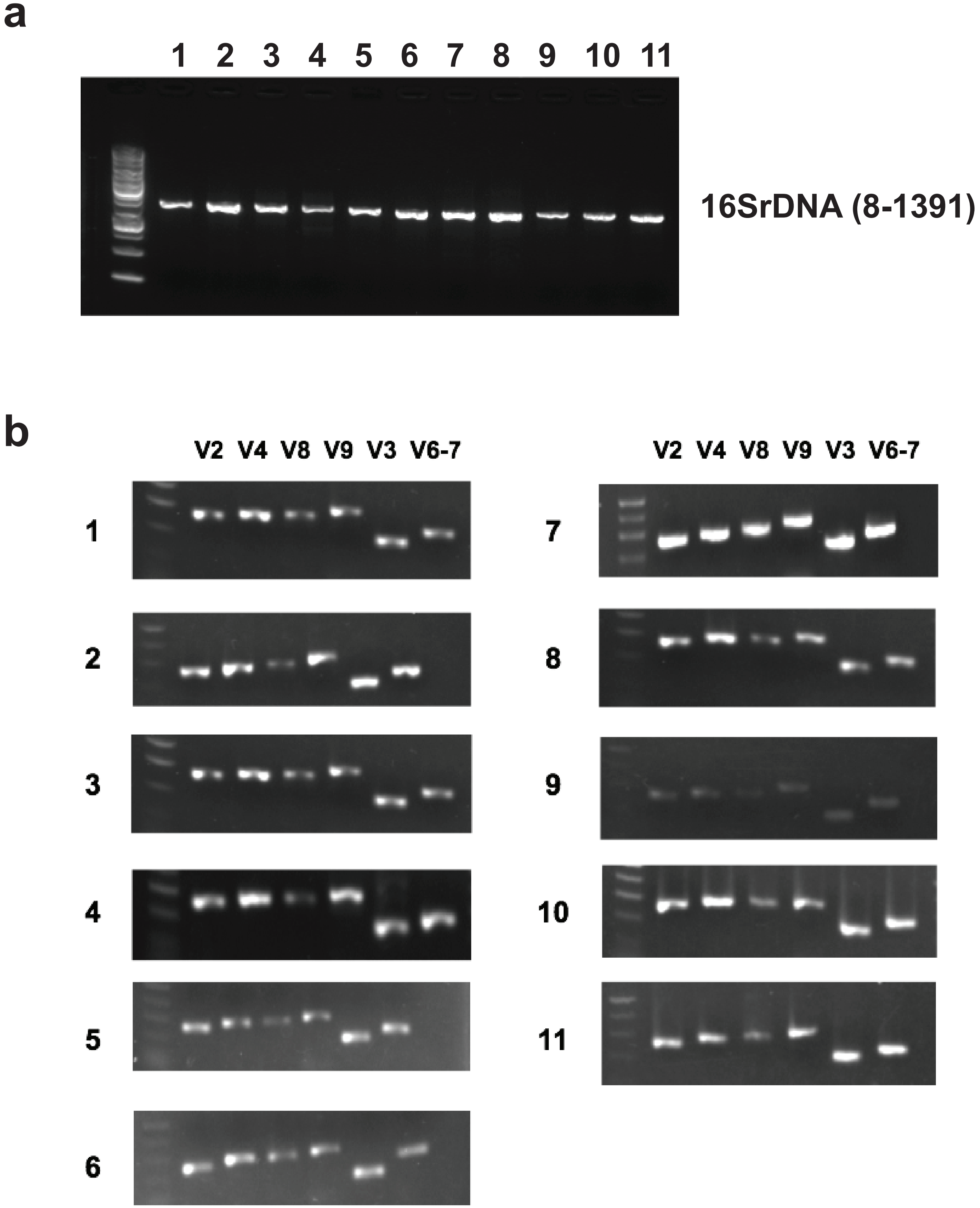

Supplemental Figure 4

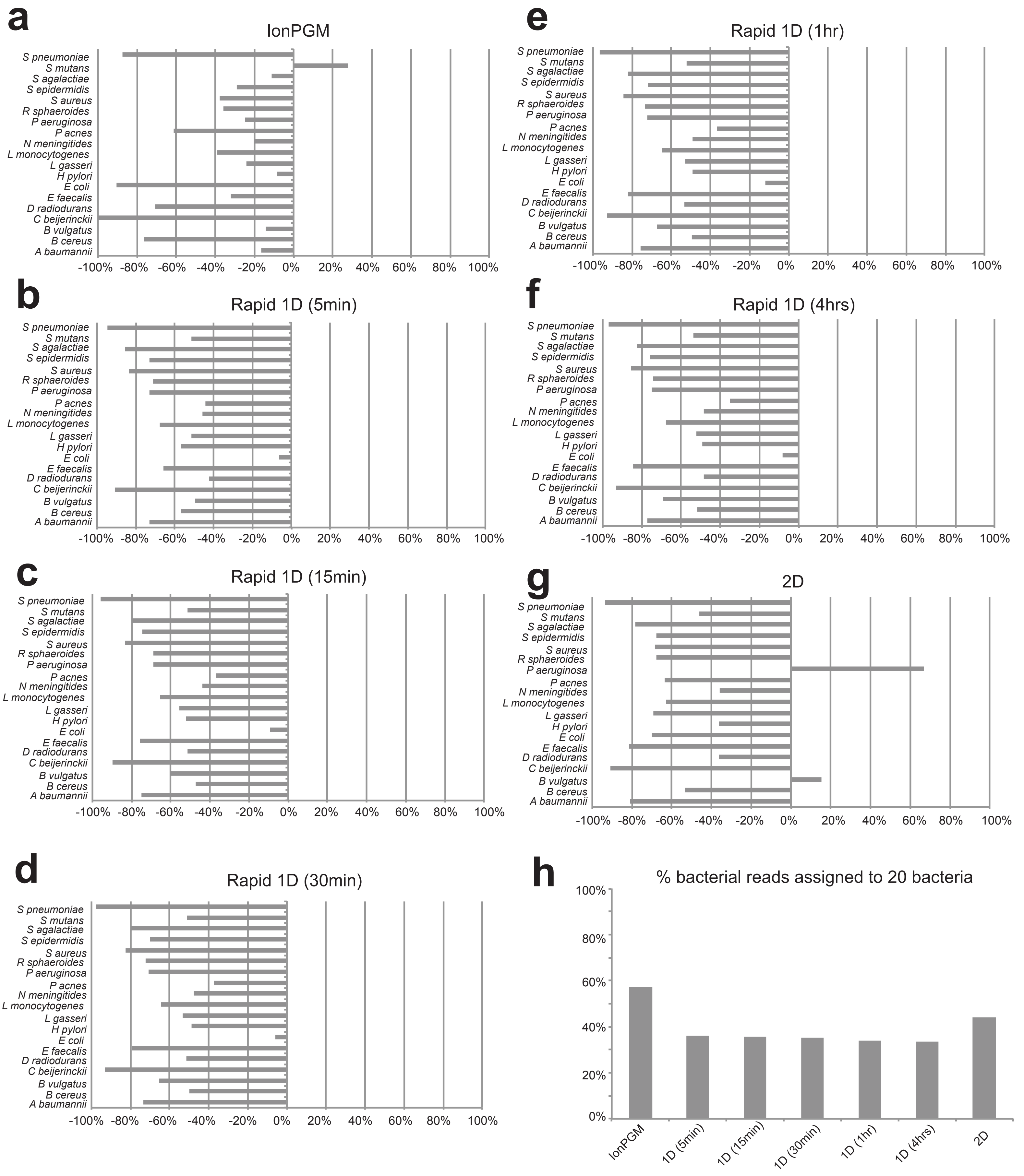

Supplemental Figure 5

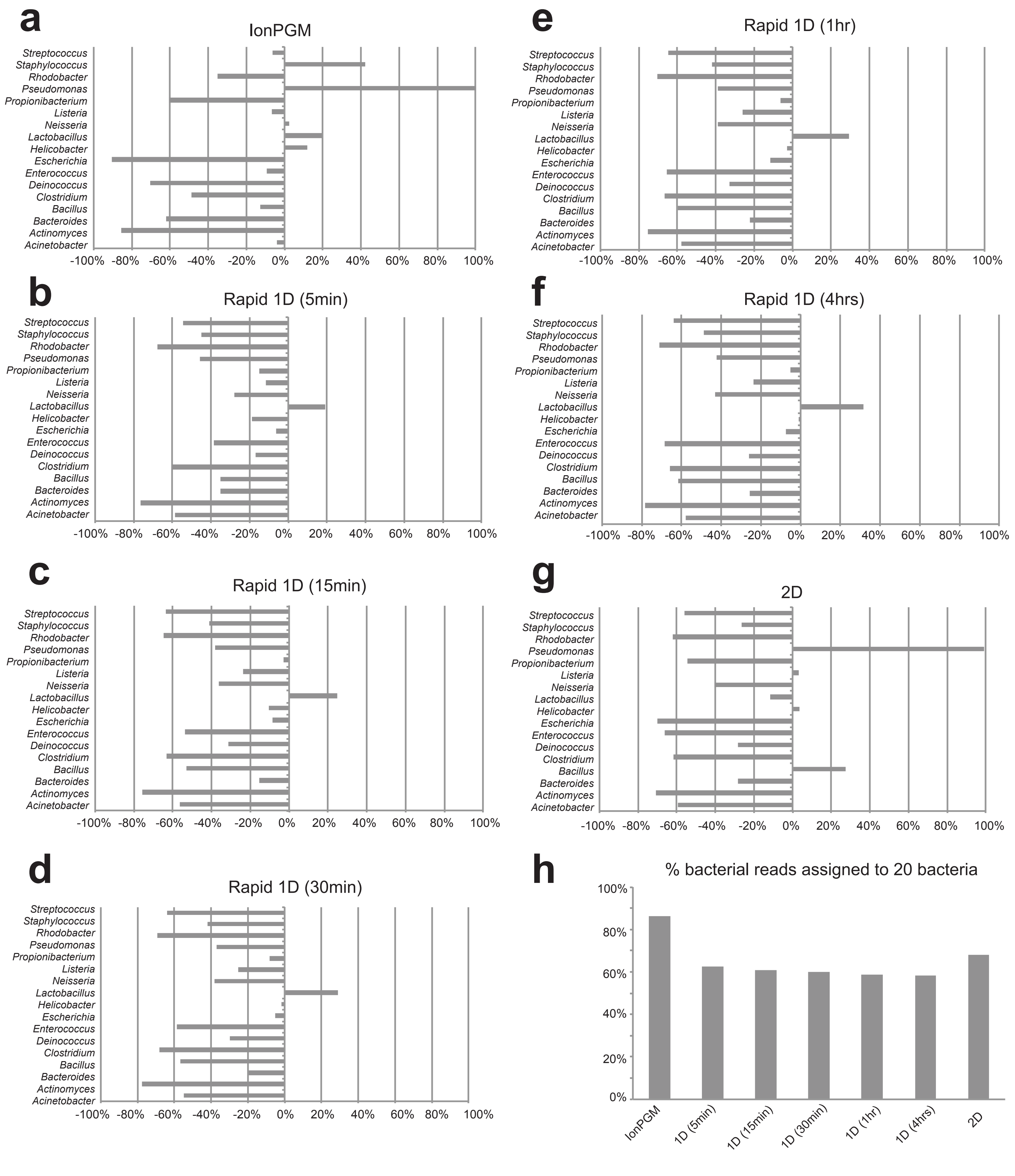

Supplemental Figure 6

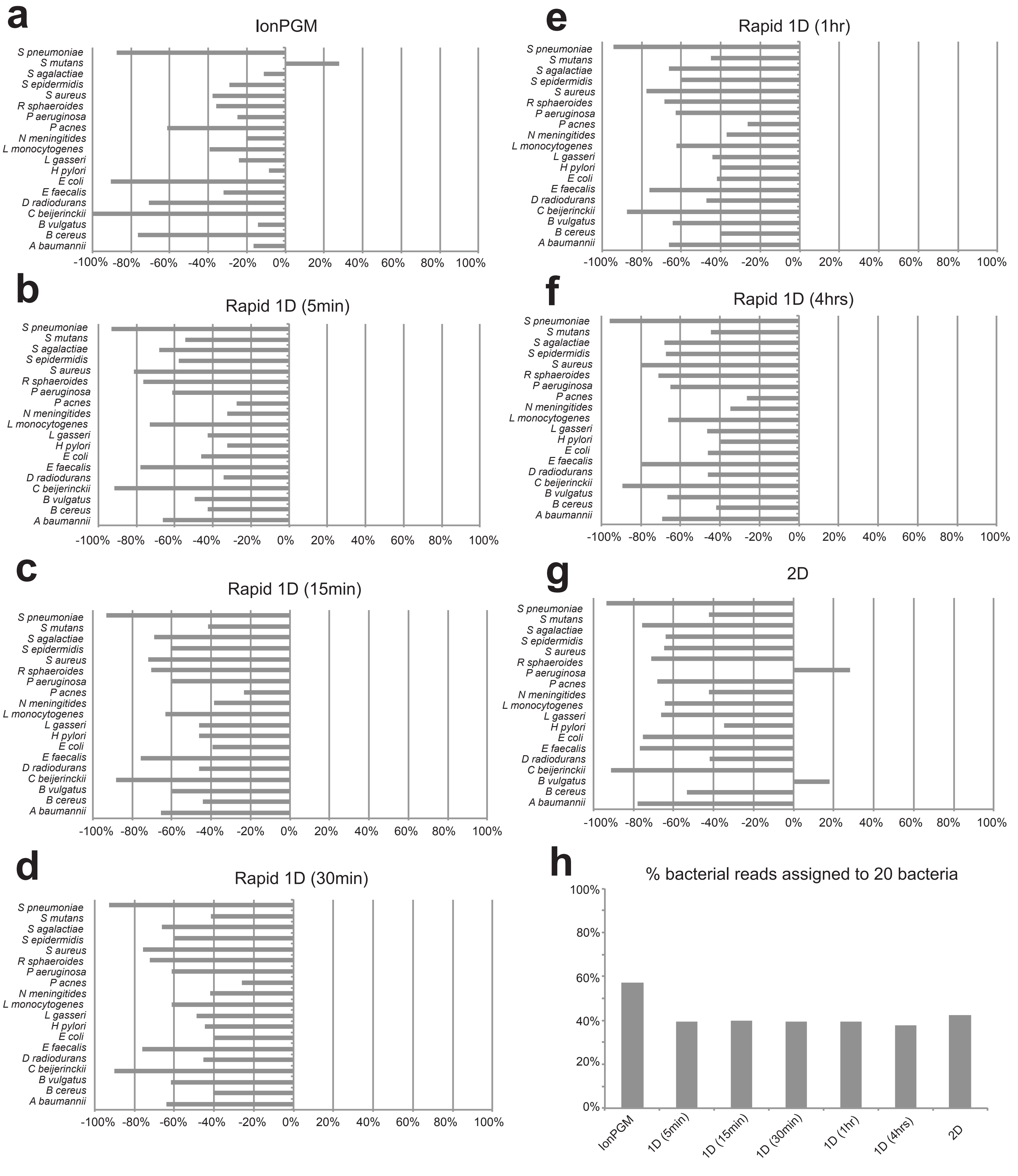

Supplemental Figure 7

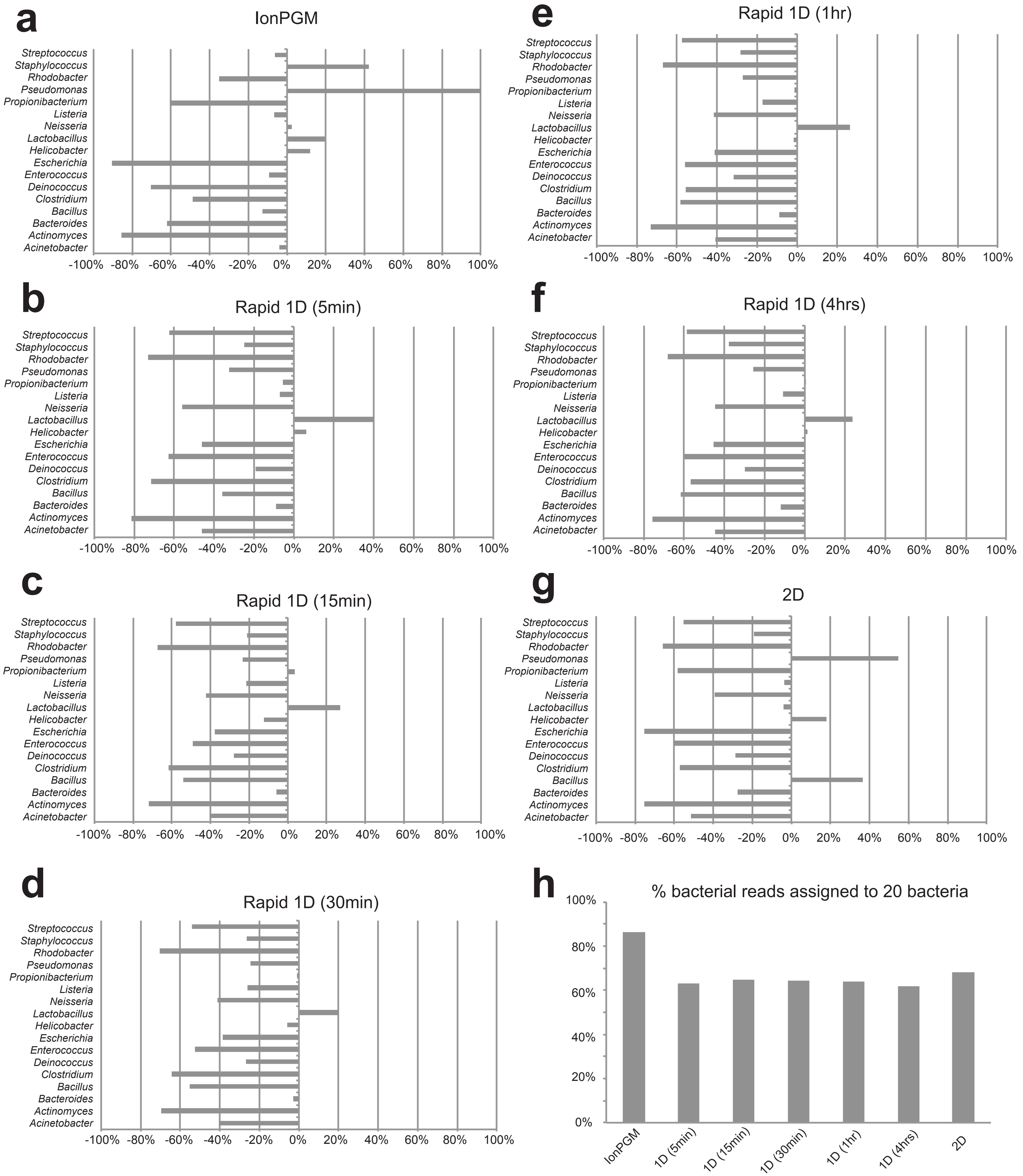

Supplemental Figure 8

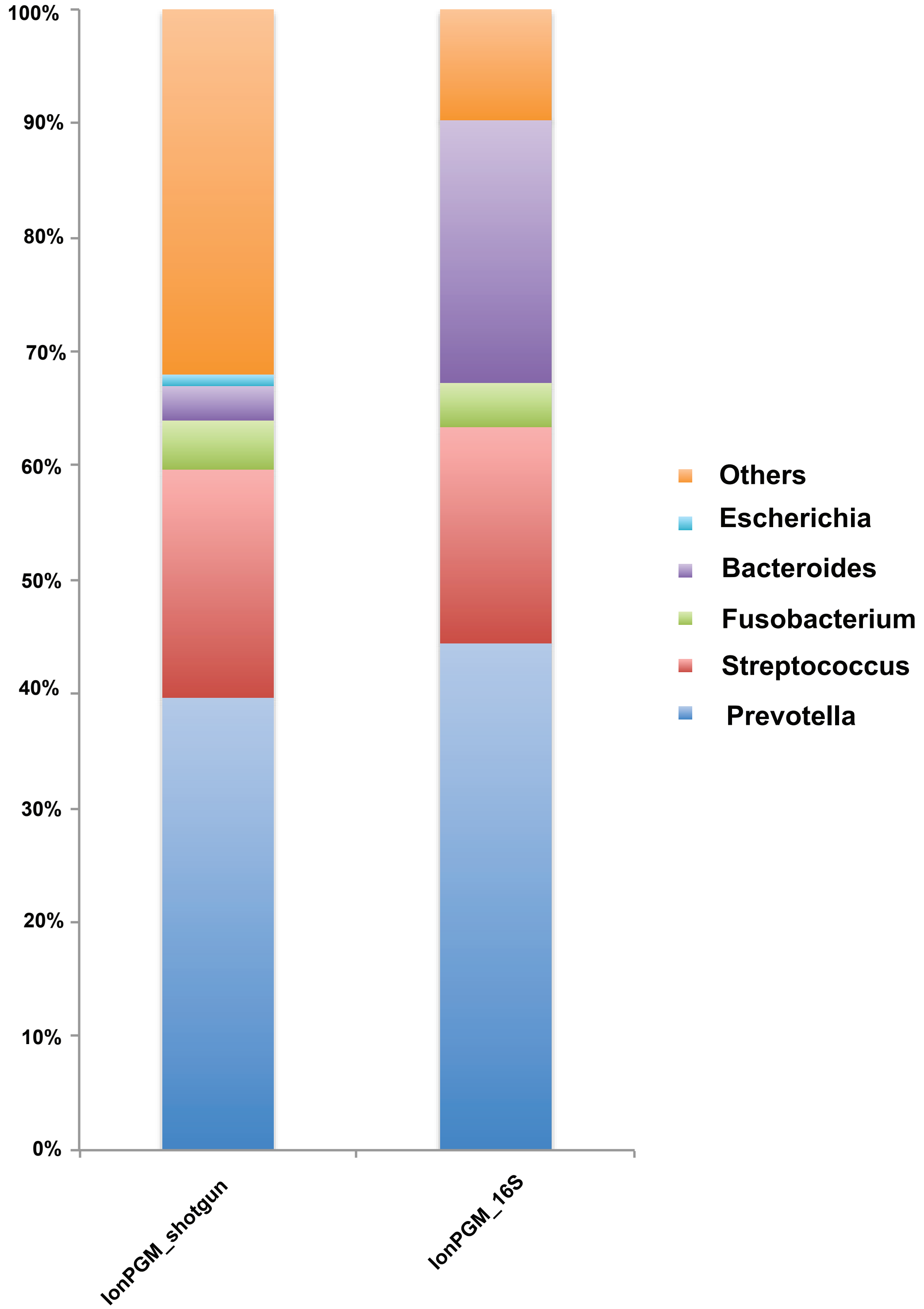

Supplement: Supplementary file 1 — Supplemental data [file 41598_2017_5772_MOESM1_ESM.pdf]
